# Psychological impact during COVID-19 pandemic: A web-based cross-sectional study among students studying at College of Science and Technology (CST), Phuentsholing, Bhutan

**DOI:** 10.1371/journal.pone.0263999

**Published:** 2022-02-17

**Authors:** Kezang Tshering, Kezang Dema

**Affiliations:** 1 Department of Pharmacy, Jigme Dorji Wangchuck National Referral Hospital, Thimphu, Bhutan; 2 Department of Information Technology, College of Science and Technology, Phuentsholing, Bhutan; National Cheng Kung University College of Medicine, TAIWAN

## Abstract

The unprecedented experience of national lockdowns and uncertainty of academic career due to the COVID-19 pandemic has multifaceted impacts on mental health among university students worldwide. This study determined its impact on depression and anxiety level, and associated risk factors among engineering students studying at College of Science and Technology (CST), Phuentsholing, Bhutan during the first lockdown in the country. Self-reported depression and anxiety levels were assessed using Patient Health Questionnaire (PHQ-9) and Generalized Anxiety Disorder-7 (GAD-7) respectively. Data was collected using an e-questionnaire link generated in Google form and the link was shared with students via the student’s official email group. A total of 278 students (response rate, 26.9%) completed the questionnaire. The majority of respondents were male (69.8%) and were aged from 18 to 30 (Mean: 21.7 ±SD 2.07) years. The prevalence of self-reported moderate to severe depression and anxiety were 44.2% (95% CI, 38.5–49.6) and 27.3% (95% CI, 22.3–32.4) respectively. Participants having their family members as frontline workers reported a significantly higher level of anxiety (χ2 = 4.85, *p* = 0.028). In multivariable logistic regression analysis, students who were academically lagging showed a higher risk of depression (AOR = 5.36, 95% CI = 2.86–10.04) and anxiety (AOR = 3.83, 95%CI = 1.86–7.88) as compared to students who were not academically behind. A high percentage of depression and anxiety was reported by students of CST during the COVID-19 pandemic. Findings from the study highlight the importance of adopting appropriate online-based teaching and learning methods to ensure timely academic and professional achievements. Moreover, the relevant stakeholders should put health system strategies in place to provide psychological support to university students during the COVID-19 pandemic.

## Introduction

The outbreak of coronavirus disease (COVID-19) was reported in December 2019 from the city of Wuhan, China [1]. COVID-19 is more contagious and spreads rapidly unlike severe acute respiratory syndrome (SARS) and the Middle East respiratory syndrome (MERS) [2]. The World Health Organization (WHO) declared COVID-19 as a global pandemic in the second week of March [3]. By the end of August 2020, more than 25.1 million people were infected causing 844,321 deaths worldwide [4].

In addition to mortality risk, COVID-19 imposed inevitable psychological distress among the general population across the globe [5, 6]. In particular, psychological impact among medical staffs [7], older adults [8], and children and adolescents [9] were reported from China during the COVID-19 outbreak. Similarly, psychological distress during the COVID-19 pandemic has been widely studied in various population. As reported in the systematic review, psychological disorder such as depression and anxiety among healthcare workers (HCWs) in African countries ranges from 9.5% to 73.3% and 12.5% to 71.9% respectively [10]. Moreover, COVID-19 frontline HCWs in Taiwan reported depressive symptoms (25.6%) and anxiety symptoms (30.6%) [11]. Further, 21.3% of Iranian HCWs reported severe and extremely severe anxiety during the COVID-19 pandemic [12]. Whereas, psychological distress among university students during the pandemic was rarely studied despite being highly vulnerable due to uncertainty of academic achievement, social life and future careers [13].

In Bhutan, the first case of COVID-19 was detected in a 76-year-old American tourist on March 6^th^, 2020 [14]. To curb the spread of COVID-19 among the Bhutanese population, the Royal Government of Bhutan (RGoB) implemented national preparedness and response plan [15]. The first-ever national lockdown was declared on August 11^th^ by the RGoB following the detection of COVID-19 in a 27-year-old Bhutanese woman who came in close contact with several people residing in four different districts in Bhutan. On August 16^th^, 2020, Phuentsholing town located in Southern Bhutan was declared as the first red zone in Bhutan after a four-year-old female was tested positive in Rt-PCR for COVID-19 from the community. Following the detection of local transmission from the community, the RGoB declared the community as a red zone enforcing complete movement restriction and surveillance within the community leading to suspension of education institutions. Such response imposed unprecedented delay and disruption of routine academic activities forcing university students to adapt to a new online-based learning system. According to WHO, the total number of confirmed cases were 228 with a mortality rate of 0% as of September 4^th^ 2020 in Bhutan [4].

Such unprecedented experience of national lockdown and uncertainty of academic career due to COVID-19 imposed multifaceted impacts on mental health among university students. Globally, the literature showed that significant proportions of university students reported depression and anxiety during the COVID-19 pandemic [16–18]. Different anxiety levels were reported by university students from Thailand, Indonesia and Taiwan [19]. However, there was a lack of study regarding the impact on mental health such as depression and anxiety among university students in Bhutan during this pandemic. Therefore, this study primarily aimed to determine the prevalence of self-reported depression and anxiety during the COVID-19 pandemic among students at the College of Science and Technology (CST), Phuentsholing located under the red zone. Moreover, we examined factors associated with depression and anxiety among our respondents to guide policy makers to put appropriate interventions in place for high-risk individuals. Although various risk factors were reported in previous studies, it differs due to cultural differences and the level of COVID-19 crisis across the country. Hence, we considered some of the variables such as students studying in the final academic year, pursuing study under government scholarship, academically lagging (having one or more backlogs), living with family members during the lockdown, tested as COVID-19 positive within the last two weeks and having family members as a frontline worker as some of the potential risk factors in our setting.

## Method

### Design

A web-based cross-sectional study was conducted among students of CST using the official email platform of students from September 10^th^ to October 10^th^, 2020. Our survey was anonymous and participants’ confidentiality was ensured.

### Setting

College of Science and Technology is in Southern Bhutan under Chhukha District providing various engineering programs in the country. It provides bachelor’s degrees in Architecture, Civil engineering, Electrical engineering, Electronics and Communication Engineering, Information Technology Engineering and Masters of Engineering in Renewable Energy. Phuentsholing was the first region to be declared as a red zone in the country after detection of the first community transmission of COVID-19 in Bhutan and residents were put under strict strategic lockdown and surveillance by the RGoB.

### Participants

Study participants included students studying at CST. Students aged ≥ 18 years and those who provided written informed consent were enrolled in the study. Students below 18 years old were excluded from the study due to inconvenience in obtaining written informed consent from their respective parents.

### Data collection procedure

An online questionnaire link generated using Google form was shared via the student’s official email on September 10^th^_,_ 2020. A questionnaire link was shared with a total of 1035 students using their official email addresses. It took approximately 15 minutes to complete the questionnaire. An electronic written informed consent was obtained from each participant before they began the survey. Data was collected using the convenience sampling method. Research Ethics Board of Health (REBH), Ministry of Health of Bhutan approved our study (REBH/Approval/2021/039), and administrative clearance was also obtained from the Dean of Research and Industrial Linkage, CST.

### Measures and covariates

Our survey questionnaire consisted of three parts. The first part obtained participant’s socio-demographic characteristics, which included sex, age, final academic year (yes vs no), financial assistance for education (government scholarship vs self), academically lagging; having one or more backlogs (yes vs no), living area during national lockdown (urban vs rural), residing with family members during lockdown (yes vs no), monthly family income, residing under red-zone (yes vs no), experienced centralized quarantine (yes vs no), tested Rt-PCR positive for COVID-19 (yes vs no), family member tested Rt-PCR positive for COVID-19 (yes vs no), exercise at home during quarantine (yes vs no) and family member a front-line worker during COVID-19 pandemic (yes vs no). Individuals who were directly involved in the control of COVID-19 in the country such as healthcare workers, police, de-suung (guardians of peace and harmony), media and essential material delivery services were considered as frontline workers in the study.

The second part of the questionnaire consisted of Patient Health Questionnaire-9 (PHQ-9) scale to assess depression among participants. Depression was categorized using PHQ-9 score as, none-minimal depression (0–4), mild depression (5–9), moderate depression (10–14), moderately severe depression (15–19) and severe depression (20–27). PHQ-9 was found to be reliable and valid in detecting the severity of depression among the general population [20, 21]. PHQ-9 score of ≥10 was considered optimum to detect depression [22].

The last part of the questionnaire included the Generalized Anxiety Disorder-7 (GAD-7) scale to assess symptoms of anxiety. Anxiety symptoms were classified using GAD-7 as, none (0–4), mild anxiety (5–9), moderate anxiety (10–14) and severe anxiety (15–21) [23]. A cut-off score of ≥10 for GAD-7 was considered the presence of anxiety among respondents in the study.

### Data analysis

Participants’ socio-demographic data were presented using descriptive statistics such as frequency and percentage. The prevalence of symptoms of depression and anxiety was determined using the aforementioned cutoff scores. Differences in prevalence of depression and anxiety among respondents with different categorical characteristics were compared using chi-square (χ2) tests. Multivariable logistic regression analysis was performed to identify risk factors associated with depression and anxiety. Adjusted odds ratio (AOR) and 95% confidence interval (95%CI) were presented to identify an association between risk factors and outcomes. The level of significance was set at *p* <0.05 and data was analyzed in Statistical Package for Social Science (SPSS) version 21.

## Results

### Socio-demographic characteristics of participants

An online questionnaire link was shared among 1035 students of CST via official student’s e-mail. Among which, 445 students opened an online questionnaire link, and 278 individuals completed the survey (26.9% response rate). The majority of our participants were male (69.8%) and were aged from 18 to 30 years (Mean: 21.7 SD ± 2.07). A majority (82.4%) of our respondents were pursuing undergraduate under full government scholarship whereas 76.3% were living with family members during the national lockdown. More than one-fourth (32.4%) of students were academically lagging as presented in Table 1.

**Table 1:** Socio-demographic characteristics of students studying at college of science and technology (n = 278).

Variables	Category	n	%
**1. Sex**	Male	194	69.8
	Female	84	30.2
**2. Age (in years)**	18–22	190	68.3
	≥23	88	31.7
**3. Are you in final academic year?**	No	195	70.1
	Yes	83	29.9
**4. Studying under government scholarship?**	No	49	17.6
	Yes	229	82.4
**5. Academically lagging** (having one or more backlogs)**?**	No	90	32.4
	Yes	188	67.6
**6. Place of residence during national lockdown**	Urban	144	51.8
	Rural	134	48.2
**7. Living with family members during national lockdown?**	No	66	23.7
	Yes	212	76.3
**8. Monthly family income**	Unknown	41	14.7
	<21,000	138	49.6
	≥21,000	99	35.6
**9. Residing at Phuentsholing town during national lockdown?**	No	203	73.0
	Yes	75	27.0
**10. Ever experienced centralized quarantine in the past 2 weeks?**	No	276	99.3
	Yes	2	0.7
**11. Tested as COVID-19 positive in RT-PCR in last 14 days?**	No	278	100.0
	Yes	0	0.0
**12. Any of your family member tested COVID-19 positive in RT-PCR?**	No	268	96.4
	Yes	10	3.6
**13. Doing any sort of physical exercise at home during quarantine?**	No	130	46.8
	Yes	148	53.2
**14. Any of your family members a front-line worker?**	No	136	48.9
	Yes	142	51.1

### Prevalence of depression and anxiety

The prevalence of depression (PHQ-9 ≥10) among our participants was 44.2% (95% CI, 38.5–49.6) with 34.2% (95% CI, 28.4–39.6) mild depression, 23.7% (95% CI, 18.7–29.1) moderate depression, 12.2% (95% CI, 8.6–16.2) moderately severe depression and 8.3% (95% CI, 5.0–11.9) severe depression. Prevalence of anxiety (GAD-7 ≥10) was 27.3% (95% CI, 22.3–32.4) with 37.4% (95% CI, 31.7–43.2) mild anxiety, 13.3% (95% CI, 9.7–17.6) moderate anxiety and 14.0% (95% CI, 10.1–18.3) severe anxiety. As shown in Table 2, Depression among students who are academically lagging was significantly higher than those who are not academically behind (χ2 = 31.71, *df* = 1, *p* <0.0005). Anxiety was reported significantly higher by students who are academically lagging (χ2 = 13.14, *df* = 1, *p* <0.0005) and students having their family members as a front-line worker (χ2 = 4.85, *df* = 1, *p* = 0.028) during COVID-19 pandemic.

**Table 2:** Level of depression and anxiety among participants with different socio-demographic characteristics.

Variables	Total	Depression		Anxiety	
	(n = 278)	(n = 123)	*p* value	(n = 76)	*p* value
**Sex**					
Male	194 (69.8)	83 (42.8)	0.456	48 (24.7)	0.140
Female	84 (30.2)	40 (47.6)		28 (33.3)	
**Age (years)**					
18–22	190 (68.3)	88 (46.3)	0.307	55 (28.9)	0.376
≥23	88 (31.7)	35 (39.8)		21 (23.9)	
**Are you in final academic year?**					
No	195 (70.1)	90 (46.2)	0.326	55 (28.2)	0.619
Yes	83 (29.9)	33 (39.8)		21 (25.3)	
**Studying under government scholarship?**					
No	49 (17.6)	26 (50.1)	0.171	13 (26.5)	0.889
Yes	229 (82.4)	97 (42.4)		63 (27.5)	
**Academically lagging** (having one or more backlogs)**?**					
No	90 (32.4)	18 (20.0)	< 0.0005	12 (13.3)	< 0.0005
Yes	188 (67.6)	105 (55.9)		64 (34.0)	
**Place of residence during national lockdown**					
Urban	144 (51.8)	67 (46.5)	0.427	36 (25.0)	0.865
Rural	134 (48.2)	56 (41.8)		40 (29.9)	
**Living with family members during national lockdown?**					
No	66 (23.7)	23 (34.8)	0.078	18 (27.3)	0.989
Yes	212 (76.3)	100 (47.2)		58 (27.4)	
**Monthly family income**					
Unknown	41 (14.7)	20 (48.8)	0.593	8 (19.5)	0.079
<21,000	138 (49.6)	63 (45.7)		46 (33.3)	
≥21,000	99 (35.6)	40 (40.4)		22 (22.2)	
**Residing at Phuentsholing town during national lockdown?**					
No	203 (73.0)	96 (47.3)	0.093	55 (27.1)	0.88
Yes	75 (27.0)	27 (36.0)		21 (28.0)	
**Ever experienced centralized quarantine in the past 2 weeks?**					
No	276 (99.3)	122 (44.2)	1.00	76 (27.5)	1.00
Yes	2 (0.7)	1 (50.0)		0 (0)	
**Tested as COVID-19 positive in RT-PCR in last 14 days?**					
No	278 (100)	-	-	76	-
Yes	0 (0)	-		0 (0)	
**Any of your family member tested COVID-19 positive in RT-PCR?**					
No	268 (96.4)	118 (44.0)	0.754	73 (27.2)	1.0
Yes	10 (3.6)	5 (50.0)		3 (30.0)	
**Doing any sort of physical exercise at home during quarantine?**					
No	130 (46.8)	61 (47.0)	0.399	37 (28.5)	0.694
Yes	148 (53.2)	62 (41.9)		39 (26.4)	
**Any of your family members a front-line worker?**					
No	136 (48.9)	57 (41.9)	0.470	29 (21.3)	0.028
Yes	142 (51.1)	66 (46.5)		47 (33.1)	

### Factors associated with depression and anxiety

Table 3 shows the risk factors of depression and anxiety among study participants. In multivariable logistic regression analysis, individuals who are academically lagging were at higher risk of self-reported mental health symptoms compared with those who are not academically behind with AOR 5.51 (95% CI 2.95–10.29) and AOR 3.64 (95% CI 1.80–7.38) for depression and anxiety respectively.

**Table 3:** Factors associated with self-reported depression and anxiety among students studying at CST during COVID-19 pandemic.

Variables	Depression		Anxiety	
	AOR (95% CI)	*p*-value	AOR (95% CI)	*p*-value
**Sex**				
Male	-		-	
Female	1.29 (0.72–2.31)	0.394	1.64 (0.89–3.05)	0.116
**Age (years)**				
18–22	-		-	
≥23	0.94 (0.49–1.78)	0.841	0.84 (0.42–1.69)	0.619
**Are you in final academic year?**				
No	-		-	
Yes	0.97 (0.45–2.12)	0.947	0.65 (0.27–1.56)	0.333
**Studying under government scholarship?**				
No	-		-	
Yes	0.70 (0.35–1.42)	0.327	1.36 (0.62–2.99)	0.441
**Academically lagging** (having one or more backlogs)**?**				
No	-		-	
Yes	5.36 (2.86–10.04)	<0.0005	3.83 (1.86–7.88)	<0.0005
**Place of residence during national lockdown**				
Urban	-		-	
Rural	0.65 (0.37–1.14)	0.134	1.40 (0.75–2.60)	0.289
**Living with family members during national lockdown?**				
No	-		-	
Yes	1.45 (0.69–3.05)	0.333	1.28 (0.57–2.86)	0.548
**Monthly family income**				
Unknown	-		-	
<21,000	1.60 (0.69–3.67)	0.272	1.98 (0.80–4.89)	0.137
≥21,000	1.40 (0.77–2.56)	0.273	0.99 (0.37–2.66)	0.984
**Residing at Phuentsholing town during national lockdown?**				
No	-		-	
Yes	0.82 (0.38–1.78)	0.608	1.53 (0.66–3.58)	0.325
**Ever experienced centralized quarantine in the past 2 weeks?**				
No	-		-	
Yes	1.39 (0.82–23.60)	0.821		
**Any of your family member tested COVID-19 positive in Rt-PCR?**				
No	-		-	
Yes	1.27 (0.30–5.33)	0.743	1.48 (0.33–6.73)	0.611
**Doing any sort of physical exercise at home during quarantine?**				
No	-		-	
Yes	0.91 (0.53–1.54)	0.721	0.87 (0.49–1.56)	0.641
**Any of your family members a front-line worker?**				
No	-		-	
Yes	1.06 (0.63–1.79)	0.829	1.67 (0.93–2.969)	0.084

## Discussion

Studies showed a high prevalence of self-reported psychological distress such as depression and anxiety among college students during the COVID-19 pandemic across the globe [24, 25]. Our study was first to assess the self-reported psychological impact and its associated risk factors among students of CST in southern Bhutan during this current pandemic. CST is the only college under the Phuentsholing region in Southern Bhutan where the first community outbreak of COVID-19 took place in the country followed by the strict strategic lockdown. Such unprecedented closure of college caused academic and professional uncertainty leading to increased risk of psychological distress among students studying at CST.

Our study found that 44.2% (95% CI, 38.5–49.6) and 27.3% (95% CI, 22.3–32.4) of students studying at CST experienced moderate-to-severe depression and anxiety respectively. Previous studies which assessed depression during the COVID-19 pandemic using the same instrument reported various rates of moderate-to-severe depression among students from Pakistan (45%) [26], China (15.58%) [27] and Bangladesh (53.8%) [18]. Studies conducted among university students using different instruments in Egypt [28], Spain [29] and Turkey [30] reported 45.9%, 34.2% and 27.1% of moderate-to-severe depression respectively. With regard to anxiety, similar studies reported moderate-to-severe anxiety ranging from 3.6% to 42.9% among college students worldwide [18, 26, 31]. Further, moderate-to-severe anxiety ranges from 12.1% to 47.1% according to previous studies which used different instruments (Depression Anxiety and Stress Scale– 21) to assess anxiety [28, 29, 32]. Such variations in prevalence of depression and anxiety could be attributed to variations in sociodemographic characteristics of study participants and instruments used to assess depression and anxiety. Moreover, such variations could be attributed to the country’s COVID-19 strategic preparedness and response plan, cultural differences, and level of COVID-19 crisis across the country. Nonetheless, the level of psychological distress reported by our participants during the COVID-19 pandemic was alarming.

Studies across the globe reported various factors associated with depression and anxiety among university students during this current pandemic. However, unlike previous studies, our study showed a higher risk of depression (AOR: 5.51, 95%CI: 2.95–10.29) and anxiety (AOR: 3.64, 95%CI: 1.80–7.38) among students who were academically lagging than their counterparts. Our finding showed that students who are academically behind anticipated failure of timely academic achievements due to unprecedented disruption of academic activities caused by the current pandemic. Since college students are at the university-to-work transition period, the COVID-19 pandemic could affect their career plans leading to greater psychological impact among respondents who are academically lagging. This underscores the importance of adopting appropriate online-based teaching and learning methodologies during the COVID-19 pandemic to ensure timely academic achievement and professional progress among engineering students of CST. Further, COVID-19 imposed digital transformation among university students where students studying in developing countries faced difficulty in such transition due to limited infrastructure as reported in the previous study [33]. As reported in the previous study, most universities lack enough resources or infrastructure to facilitate online-based teaching immediately [34]. Similarly, not being able to facilitate appropriate online-based teaching with immediate effect due to scarcity of infrastructure and resources at CST could have triggered greater psychological distress among students who are academically behind. Our study showed that depression and anxiety were not associated with the monthly income of family. On contrary, both depression and anxiety were associated with the low economic level of the family in the previous study [35]. This could be attributed to the majority (82.4%) of our respondents pursuing their studies under government scholarship unlike students in developed countries.

Additionally, respondents having their family members as front-line workers during the COVID-19 pandemic reported a significantly higher level of anxiety in the study (*p* = 0.028). This indicates that front-line workers being at increased risk of acquiring COVID-19 infection precipitated a high level of anxiety among their family members. A similar finding was reported in China [36].

Cross-sectional study design, failure to include other university students in Bhutan during the COVID-19 pandemic as a comparison group, not being able to establish a causal relationship of depression and anxiety with academically lagging and failure to use specific instruments for COVID-19 such as coronavirus anxiety scale (CAS) are some of the major limitations of the study.

## Conclusion

Despite limitations, our study was the first of a kind to assess the psychological impact among college-going students during the COVID-19 pandemic in Bhutan. A large percentage of students studying at CST reported depression and anxiety. Findings from the study underscore the importance of adopting appropriate online-based teaching and learning methods focused on timely academic achievements for students of CST during the COVID-19 pandemic. In addition, the relevant stakeholders should put mental health services in place to support college students in fighting against psychological distress during the COVID-19 pandemic. Further, parents should avoid imposing academic pressure among college students unless the students are comfortable with the new teaching and learning environment during the current pandemic.

## Supporting information

S1 Dataset (XLSX)
